# 

**Published:** 1848

**Authors:** 


### CONTENTS.

### PART I.—ORIGINAL COMMUNICATIONS.

ART. 1. Medical Topography of Green Castle, Indiana, and vicinity. By R. Curran, M. D., 275

ART. 2. Hernia Cerebri. Extracts of a paper read before the Indianapolis Medical Society. By G. W. Mears, M. D., 282

ART. 3. Abdominal Movements with reference to Pregnancy—their nature and cause. By J. E. McGirr, A. M., M. D., Professor of Anatomy, Physiology, and Chemistry, in the University of the Lake, Chicago, 287

ART. 4. Case of Cerebral Abscess. By Thos. Hall, M. D., Toulon, Ind., 291

ART. 5. Poisoning by Coculus Indicus. By H. Rosenkrans, M. D., 295

ART. 6. A new Treatment of Placenta Prævia. By H. Huber, M. D. ,296

ART. 7. A Case of Neuralgia. By J. George Osbourne, M. D., of Tippecanoe county, Ind., 298

### PART II.—REVIEWS AND NOTICES OF NEW WORKS.

ART. 1. A Practical Treatise on the Diseases of Children. By J. Forsyth Meigs, M. D., &c. Philadelphia: Lindsay & Blakiston. 1848. pp. 575 8vo. 300

ART. 2. A System of Human Anatomy, General and Special, By Erasmus Wilson, M. D., Lecturer on Anatomy, London. Fourth American, from the last London edition. Edited by Paul B. Goddard, A. M., M. D., Professor of Anatomy and Histology in the Franklin Medical College of Philadelphia. Philadelphia: Lea & Blanchard. 1848. pp. 376, 322

ART. 3. On Bandaging and other Operation of Minor Surgery. By F. W. Sargent, M. D. Philadelphia: Lea & Blanchard, 1848. pp. 379, 322

### PART III.—SELECTIONS.

ART. 1. On certain Forms of Headache. By Dr. Murphy. 324

ART. 2 On the Medical Treatment of Cataract. By Wm. G. Smith, M. D. Communicated, by letter to Jas. Bryan, M. D., to the College of Physicians and Surgeons of Philadelphia, 327

ART. 3. Case of Hœmaturia—Death—Carcinoma of Bladder. By Austin Flint, M. D., Professor of Principles and Practice of Medicine in the University of Buffalo, 332

ART. 4. Anti-Mercury and Anti-Mineral Doctors, 334

ART. 5. Anthropo-toxicologia. Cases; with remarks. Read before the Alabama Medical Society, April 3, 1848. By C. E. Lavender, M. D., of Salem, Alabama, 339

ART. 6. A Contribution to the Curiosities of Medical Experience. By T. M. Harris, M. D., of Harrisville, Va., 345

ART. 7. A Statement in relation to the United States Naval Medical Corps, 344

ART. 8. The Anodyne Treatment in Croup, 350

ART. 9. The late Baron Berzelius, 352

ART. 10, New Medical School, 356

ART. 11. The Cholera, 356

ART. 11. Cholera in Granite Regions, 357

ART. 12. On the Use of Solution of Caustic Potash in relieving Strangury. By R. Mulock, M. D., 358

ART. 13. Pathology of Intermittent Diseases, 359

ART. 14. On the Internal Use of Turpentine Oil in Cases of Hemorrhage. By L. Percy, M. D., 360

ART. 15. Remarks on the Bearing of some Modern Doctrines of Pathology and Animal Chemistry on the Treatment of Tubercular Consumption.—By E. J. Marsh, M. D., 361

### PART IV.—EDITORIALS.

ART. 1. New Medical Schools, 364

ART. 2. Convention of Delegates from Western Medical Schools, 366

ART. 3. Medical Miscellany. 367

